# Outcome of Hospitalization for COVID-19 in Patients with Interstitial Lung Disease. An International Multicenter Study

**DOI:** 10.1164/rccm.202007-2794OC

**Published:** 2020-12-15

**Authors:** Thomas M. Drake, Annemarie B. Docherty, Ewen M. Harrison, Jennifer K. Quint, Huzaifa Adamali, Sarah Agnew, Suresh Babu, Christopher M. Barber, Shaney Barratt, Elisabeth Bendstrup, Stephen Bianchi, Diego Castillo Villegas, Nazia Chaudhuri, Felix Chua, Robina Coker, William Chang, Anjali Crawshaw, Louise E. Crowley, Davinder Dosanjh, Christine A. Fiddler, Ian A. Forrest, Peter M. George, Michael A. Gibbons, Katherine Groom, Sarah Haney, Simon P. Hart, Emily Heiden, Michael Henry, Ling-Pei Ho, Rachel K. Hoyles, John Hutchinson, Killian Hurley, Mark Jones, Steve Jones, Maria Kokosi, Michael Kreuter, Laura S. MacKay, Siva Mahendran, George Margaritopoulos, Maria Molina-Molina, Philip L. Molyneaux, Aiden O’Brien, Katherine O’Reilly, Alice Packham, Helen Parfrey, Venerino Poletti, Joanna C. Porter, Elisabetta Renzoni, Pilar Rivera-Ortega, Anne-Marie Russell, Gauri Saini, Lisa G. Spencer, Giulia M. Stella, Helen Stone, Sharon Sturney, David Thickett, Muhunthan Thillai, Tim Wallis, Katie Ward, Athol U. Wells, Alex West, Melissa Wickremasinghe, Felix Woodhead, Glenn Hearson, Lucy Howard, J. Kenneth Baillie, Peter J. M. Openshaw, Malcolm G. Semple, Iain Stewart

**Affiliations:** ^1^ Centre for Medical Informatics, The Usher Institute, University of Edinburgh, Edinburgh, United Kingdom; ^2^ National Heart and Lung Institute, Imperial College, London, United Kingdom; ^3^ Bristol Interstitial Lung Disease Service, North Bristol NHS Trust and; ^4^ Academic Respiratory Unit, University of Bristol, Southmead Hospital, Bristol, United Kingdom; ^5^ Liverpool Interstitial Lung Disease Service, Aintree site, Liverpool University Hospitals NHS Foundation Trust, Liverpool, United Kingdom; ^6^ Queen Alexandra Hospital, Portsmouth, United Kingdom; ^7^ Northern General Hospital, Sheffield, United Kingdom; ^8^ Centre for Rare Lung Diseases, Department of Respiratory Diseases and Allergy, Aarhus University Hospital, Aarhus, Denmark; ^9^ Interstitial Lung Disease (ILD) Unit, Respiratory Medicine Department, Hospital of the Holy Cross and Saint Paul, Barcelona, Spain; ^10^ ILD Unit, Manchester University Hospital NHS Foundation Trust, Wythenshawe Hospital, Wythenshawe, United Kingdom; ^11^ University of Manchester, Manchester, United Kingdom; ^12^ Royal Brompton and Harefield NHS Foundation Trust, London, United Kingdom; ^13^ Respiratory Medicine, Hammersmith Hospital, Imperial College Healthcare NHS Trust, London, United Kingdom; ^14^ Nottingham University Hospitals NHS Trust, Nottingham, United Kingdom; ^15^ Birmingham Interstitial Lung Disease Unit, Queen Elizabeth Hospital Birmingham, University Hospitals Birmingham NHS Foundation Trust, Birmingham, United Kingdom; ^16^ University of Birmingham, Birmingham, United Kingdom; ^17^ Cambridge Interstitial Lung Disease Service, Royal Papworth Hospital NHS Foundation Trust, Cambridge, United Kingdom; ^18^ Department of Respiratory Medicine, Royal Victoria Infirmary, Newcastle upon Tyne Hospitals NHS Foundation Trust, Newcastle upon Tyne, United Kingdom; ^19^ South West Peninsula ILD Network, Royal Devon & Exeter Foundation NHS Trust, Exeter, United Kingdom; ^20^ Northumbria Specialist Emergency Care Hospital, Northumbria Healthcare NHS Foundation Trust, Cramlington, United Kingdom; ^21^ Respiratory Research Group, Hull York Medical School, Castle Hill Hospital, Cottingham, United Kingdom; ^22^ University Hospitals Southampton NHS Foundation Trust, Southampton, United Kingdom; ^23^ Cork University Hospital, Cork, Ireland; ^24^ Oxford Interstitial Lung Disease Service, Oxford University Hospitals NHS Foundation Trust, Oxford, United Kingdom; ^25^ King’s Mill Hospital, Nottinghamshire, United Kingdom; ^26^ Department of Medicine, Royal College of Surgeons in Ireland, Dublin, Ireland; ^27^ Beaumont Hospital, Dublin, Ireland; ^28^ National Institute for Health Research (NIHR) Southampton Biomedical Research Centre & Clinical and Experimental Sciences, University of Southampton, Southampton, United Kingdom; ^29^ Action for Pulmonary Fibrosis, Stuart House, Peterborough, United Kingdom; ^30^ Guys and St. Thomas’ NHS Trust, London, United Kingdom; ^31^ Center for Interstitial and Rare Lung Diseases, Pneumology, Thoraxklinik, University of Heidelberg and German Center for Lung Research, Heidelberg, Germany; ^32^ Kingston Hospital NHS Foundation Trust, Surrey, United Kingdom; ^33^ ILD Unit, Respiratory Department, University Hospital of Bellvitge, Institut d’Investigació Biomèdica de Bellvitge, Hospitalet de Llobregat, Barcelona, Spain; ^34^ University Hospital Limerick, Limerick, Ireland; ^35^ Department of Respiratory Medicine, Mater Misericordiae University Hospital, Dublin, Ireland; ^36^ Department of Diseases of the Thorax, Morgagni Hospital, Forli, Italy; ^37^ UCL Respiratory, University College London and ILD Service, University College London Hospitals NHS Foundation Trust, London, United Kingdom; ^38^ Imperial Healthcare NHS Trust, St. Mary’s Hospital, The Bays, London, United Kingdom; ^39^ Laboratory of Biochemistry and Genetics, Pneumology Unit, Fondazione Istituto di Ricovero e Cura a Carattere Scientifico Policlinico San Matteo, Pavia, Italy; ^40^ University Hospital North Midlands NHS Trust, Royal Stoke University Hospital, Stoke-on-Trent, United Kingdom; ^41^ Royal United Hospitals Bath NHS Foundation Trust, Bath, United Kingdom; ^42^ Institute of Lung Health, Interstitial Lung Disease Unit, Glenfield Hospital, Leicester, United Kingdom; ^43^ NIHR Biomedical Research Centre, Respiratory Research Unit, University of Nottingham, Nottingham, United Kingdom; ^44^ Roslin Institute, University of Edinburgh, Edinburgh, United Kingdom; ^45^ Intensive Care Unit, Royal Infirmary Edinburgh, Edinburgh, United Kingdom; ^46^ NIHR Health Protection Research Unit in Emerging and Zoonotic Infections, Institute of Infection, Veterinary and Ecological Sciences, Faculty of Health and Life Sciences, University of Liverpool, Liverpool, United Kingdom; and; ^47^ Respiratory Medicine, Alder Hey Children’s Hospital, Liverpool, United Kingdom

**Keywords:** COVID-19, idiopathic pulmonary fibrosis, interstitial lung disease, obesity, lung function

## Abstract

**Rationale:** The impact of coronavirus disease (COVID-19) on patients with interstitial lung disease (ILD) has not been established.

**Objectives:** To assess outcomes in patients with ILD hospitalized for COVID-19 versus those without ILD in a contemporaneous age-, sex-, and comorbidity-matched population.

**Methods:** An international multicenter audit of patients with a prior diagnosis of ILD admitted to the hospital with COVID-19 between March 1 and May 1, 2020, was undertaken and compared with patients without ILD, obtained from the ISARIC4C (International Severe Acute Respiratory and Emerging Infection Consortium Coronavirus Clinical Characterisation Consortium) cohort, admitted with COVID-19 over the same period. The primary outcome was survival. Secondary analysis distinguished idiopathic pulmonary fibrosis from non–idiopathic pulmonary fibrosis ILD and used lung function to determine the greatest risks of death.

**Measurements and Main Results:** Data from 349 patients with ILD across Europe were included, of whom 161 were admitted to the hospital with laboratory or clinical evidence of COVID-19 and eligible for propensity score matching. Overall mortality was 49% (79/161) in patients with ILD with COVID-19. After matching, patients with ILD with COVID-19 had significantly poorer survival (hazard ratio [HR], 1.60; confidence interval, 1.17–2.18; *P* = 0.003) than age-, sex-, and comorbidity-matched controls without ILD. Patients with an FVC of <80% had an increased risk of death versus patients with FVC ≥80% (HR, 1.72; 1.05–2.83). Furthermore, obese patients with ILD had an elevated risk of death (HR, 2.27; 1.39−3.71).

**Conclusions:** Patients with ILD are at increased risk of death from COVID-19, particularly those with poor lung function and obesity. Stringent precautions should be taken to avoid COVID-19 in patients with ILD.

**At a Glance Commentary**

**Scientific Knowledge on the Subject**

The outcome of patients with interstitial lung disease (ILD) admitted to the hospital with coronavirus disease (COVID-19) infection and the risk factors associated with severe COVID-19 are not known.

**What This Study Adds to the Field**

The study demonstrates that patients with ILD are at higher risk of death after COVID-19 than matched patients without ILD, and the risk factors for death include male sex, an FVC <80% predicted, and obesity.

Interstitial lung diseases (ILDs) represent a group of fibroinflammatory diseases affecting the alveolar interstitium of the lung. ILDs are characterized by alveolar damage and interstitial thickening and, if left untreated, lead to remorseless progression of breathlessness, cough, and, ultimately, death from respiratory failure. The prevalence of ILDs in Europe is just under 1 per 1,000 people, with an annual incidence of approximately 20 per 100,000 people (1). The commonest ILD is sarcoidosis, but one of the most severe ILDs is idiopathic pulmonary fibrosis (IPF), a high incidence of which is found in the UK (12 per 100,000) (2, 3). IPF tends to affect older people, with a mean age at diagnosis of 72 years. Men are more often affected than women. Furthermore, IPF is associated with diabetes mellitus type 2, hypertension, and ischemic heart disease (4, 5). People suffering from other ILDs (non-IPF ILD) tend to be younger, with a higher proportion of female sufferers, and often receive immunosuppressive therapy.

All ILDs, most notably IPF, are characterized by acute exacerbations, which have a particularly high mortality rate ranging from 35% to 70% (6). The precise cause of acute exacerbations is unknown, but they have been associated with thoracic surgical procedures and viral infections (6). Furthermore, an acute exacerbation is associated with the development of acute respiratory distress syndrome (ARDS), which carries a high mortality and morbidity. However, there is no consensus on treatment of ARDS in this group of patients, given that mechanical ventilation is the cornerstone of supportive therapy in patients without ILD (7).

Infection with severe acute respiratory syndrome coronavirus 2 (SARS-CoV-2) may lead to coronavirus disease (COVID-19), characterized by a severe viral pneumonia and ARDS in approximately 20% of patients admitted to the hospital (8). Patients most at risk of severe COVID-19 include elderly males with comorbidities including diabetes mellitus type 2, hypertension, and ischemic heart disease (9, 10), which are shared by patients with ILD, most notably IPF (5). To understand the risk to patients with ILD hospitalized with COVID-19 and therefore the potential benefits of self-isolation during the pandemic, we undertook an international multicenter analysis of patients admitted to the hospital between March 1 and May 1, 2020, with COVID-19. We compared outcomes of patients with ILD with age-, sex-, and comorbidity-matched controls admitted with COVID-19 but without ILD, from a prospective UK cohort, the ISARIC4C CCP-UK (International Severe Acute Respiratory and Emerging Infection Consortium Coronavirus Clinical Characterisation Protocol UK).

Some of the results of these studies have been previously reported in the form of a preprint (https://doi.org/10.1101/2020.07.15.20152967).

## Methods

### Patients

Physicians admitting patients with ILD throughout Europe were contacted through research networks, e-mail contacts, and social media and asked to identify all patients with a preexisting diagnosis of ILD admitted to the hospital between March 1 and May 1, 2020, during the first peak of the COVID-19 pandemic. Participating centers included tertiary ILD centers from Denmark, Germany, Italy, the Republic of Ireland, Spain, and the UK and secondary care hospitals from the Republic of Ireland and the UK to obtain as representative a European ILD population as possible. Deidentified, unlinked data from individuals were included in the audit. Each contributing site was asked to identify individuals admitted to their local hospital. As SARS-CoV-2 was an emerging virus at the start of the pandemic and PCR testing has a high false-negative rate, we defined the diagnosis of COVID-19 based on a SARS-CoV-2–positive PCR swab and/or clinicoradiological diagnosis. Audit data were unified with the ISARIC4C CCP-UK database, with separate categorization of individuals who reported an existing chronic pulmonary disease. The ISARIC4C CCP-UK database is a prospective cohort study enrolling patients across the UK admitted to the hospital with COVID-19. Patients admitted were identified by local investigators and followed up prospectively by clinical research staff. Detailed data on comorbidities, treatments received, and outcomes were captured. The protocol is available at https://isaric.net/ccp/.

Data on the presence of chronic lung disease and asthma and prescription data were collected for the comparator group. Based on these variables, patients with no previous respiratory disease were selected to most accurately estimate the change in hazard for patients with ILD. Significant comorbidities of diabetes (type 1 or type 2), hypertension, chronic heart disease, and malignancy, as well as immunomodulatory therapies, were recorded in both ILD audit data and the ISARIC database.

### Outcomes

The primary outcome was in-hospital mortality, and other recorded outcomes were whether the patient was discharged or remained hospitalized at the date of censoring (May 1, 2020). Secondary outcomes included whether patients were ventilated or received continuous positive airway pressure, other noninvasive ventilation, or high-flow oxygen and were described as “enhanced respiratory support.” Length of stay was recorded at date of death, discharge, or date of censoring. All outcomes were recorded on a standardized case report form and entered without any identifiers directly onto a secure REDcap database.

### Ethics and Consent

All data were entered by the local clinical care team in anonymized fashion without linkage to any patient identifiers in line with national and local audit guidance. In the UK, Health Research Authority guidance was followed (https://www.hra.nhs.uk/covid-19-research/guidance-using-patient-data/), and ethical approval was not required. Similar regulations applied in Denmark, Italy, and the Republic of Ireland. ISARIC4C CCP-UK received ethical approval from the South Central - Oxford C Research Ethics Committee in England (Ref: 13/SC/0149), and by the Scotland A Research Ethics Committee (Ref: 20/SS/0028). Data from Spain were collected under the approval for observational studies (Ref: UIC-IBU-2020-03 and PR217/20). Data from Germany were collected under approval from ethics committee of the Medical faculty of the University of Heidelberg (Ref: S-186/2020).

### Statistical Analysis

Summary statistics are presented as frequencies and percentages for categorical data and mean (SD) for normally distributed continuous data. Where continuous data were not normally distributed, the median (interquartile range [IQR]) was used. Differences in categorical data were compared using the chi-square test or Fisher’s exact test when expected counts were below 5 in any group. For continuous normally distributed two-group data, we compared differences using Welch’s *t* test or Mann-Whitney *U* if data were not normally distributed. For multiple group comparisons of continuous data, we used Kruskal-Wallis tests. Data from the ILD data set were matched in a 1:2 ratio with patients who did not have ILD from the ISARIC4C CCP-UK data set using a nearest neighbor propensity score matching algorithm. Patients in ISARIC4C CCP-UK with ILD were excluded to avoid double counting. We matched on confounders known to affect outcomes from COVID-19 disease including age, sex, diabetes, chronic cardiac disease, cancer, hypertension, and renal disease. Unmatched and matched populations were compared using standardized mean differences, plots, and summary statistics. For survival analyses, time was taken as admission to death (in-hospital survival) using length of stay where date was not available. Discharge from hospital was considered an absorbing state (once discharged, patients were considered no longer at risk of death). Discharged patients were not censored and included in the risk set until the end of follow-up; thus, discharge did not compete with death. We generated subclasses based on the propensity score distance and included these as terms within Cox proportional hazards models, where estimates are presented as hazard ratios (HRs), alongside the corresponding 95% confidence interval (CI) (11). Data were analyzed using R version 3.6.3 (R Foundation for Statistical Computing) with tidyverse, matchit, and finalfit packages.

## Results

### Baseline Demographics

**Figure 1.**
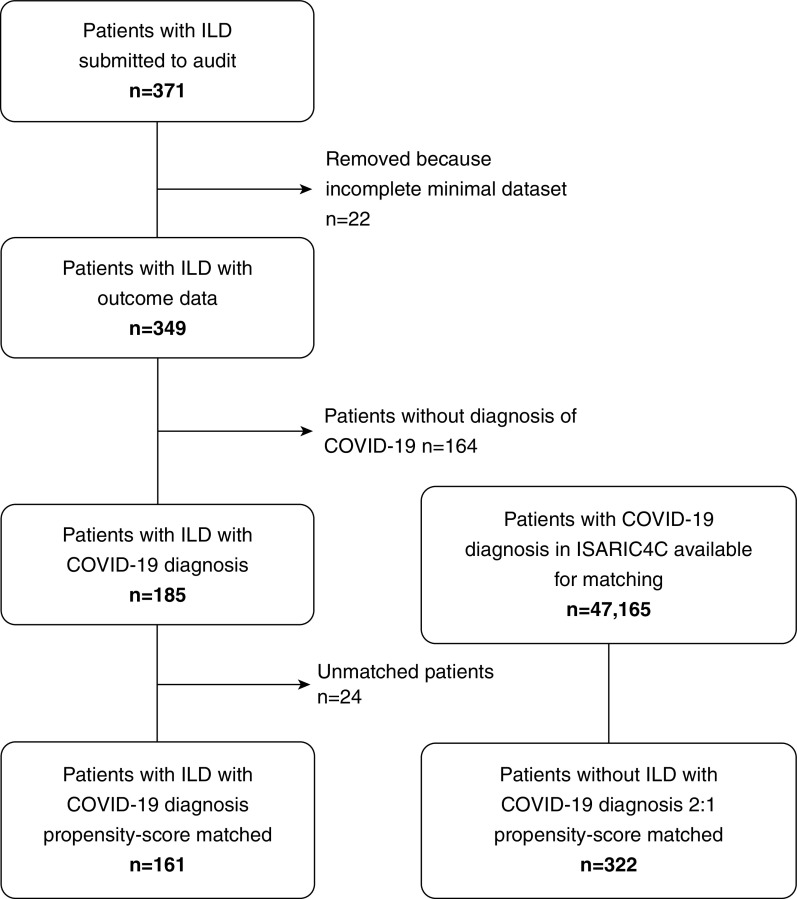
Audit flow diagram showing patients recruited and flow to matching. COVID-19 = coronavirus disease; ILD = interstitial lung disease; ISARIC4C = International Severe Acute Respiratory and Emerging Infection Consortium Coronavirus Clinical Characterisation Consortium.

Between March 1 and May 1, 2020, 349 patients with ILD were admitted to 37 hospitals throughout the UK and Europe (*see* Table E1 in the online supplement). A total of 185 patients with ILD had a diagnosis of COVID-19, and 161 were suitable for propensity score matching (Figure 1). The majority of these 161 patients (114) had a positive SARS-CoV-2 PCR test, and only 47 were diagnosed clinically. Of these, 110 (68%) were male and the mean age was 73.2 (11.5) years. The most common ILD was IPF, with 68 (42%) patients admitted to the hospital (Table 1). During the same period, 164 patients with ILD were admitted with an alternative non-COVID diagnosis, of whom 69 (42.1%) had IPF. Overall, the in-hospital mortality for patients with ILD and COVID-19 was 49% (79/161) compared with 17% (28/164) for patients with ILD admitted for other reasons.

**Table 1.** Patient Characteristics by ILD

	Total (*n*)	No ILD	ILD	*P* Value
Total	483	322 (66.7)	161 (33.3)	—
Age, yr, mean (SD)	483	72.6 (13.4)	73.2 (11.5)	0.639*
Age categories	483			
<50 yr		10 (3.1)	5 (3.1)	1.000
50–69 yr		112 (34.8)	56 (34.8)	—
70–79 yr		96 (29.8)	48 (29.8)	—
≥80 yr		104 (32.3)	52 (32.3)	—
Sex at birth, M	483	220 (68.3)	110 (68.3)	1.000
ILD				
Chronic hypersensitivity pneumonitis		0 (0.0)	14 (8.7)	—
Connective tissue disease–related ILD		0 (0.0)	13 (8.1)	—
Idiopathic pulmonary fibrosis		0 (0.0)	68 (42.2)	—
Other		0 (0.0)	47 (29.2)	—
Rheumatoid-related ILD		0 (0.0)	10 (6.2)	—
Sarcoidosis		0 (0.0)	9 (5.6)	—
Chronic cardiac disease, yes	483	92 (28.6)	46 (28.6)	1.000
Diabetes, yes	483	98 (30.4)	49 (30.4)	1.000
Malignant neoplasm, yes	483	56 (17.4)	28 (17.4)	1.000
Obesity	419			
Yes		23 (7.1)	45 (28.0)	<0.001
Missing		32 (9.9)	32 (19.9)	—
Hypertension, yes	483	148 (46.0)	74 (46.0)	1.000
Chronic kidney disease, yes	483	16 (5.0)	8 (5.0)	1.000
Pirfenidone, yes	483	0 (0.0)	7 (4.3)	—
Nintedanib, yes	483	0 (0.0)	13 (8.1)	—
Corticosteroids, yes	483	28 (8.7)	45 (28.0)	<0.001

*Definition of abbreviation*: ILD = interstitial lung disease.

Data are shown as *n* (%) unless noted otherwise. All tests are chi-square except where noted.

* Welch’s two-sample *t* test used.

### Effect of ILD on Outcome from COVID-19

**Figure 2.**
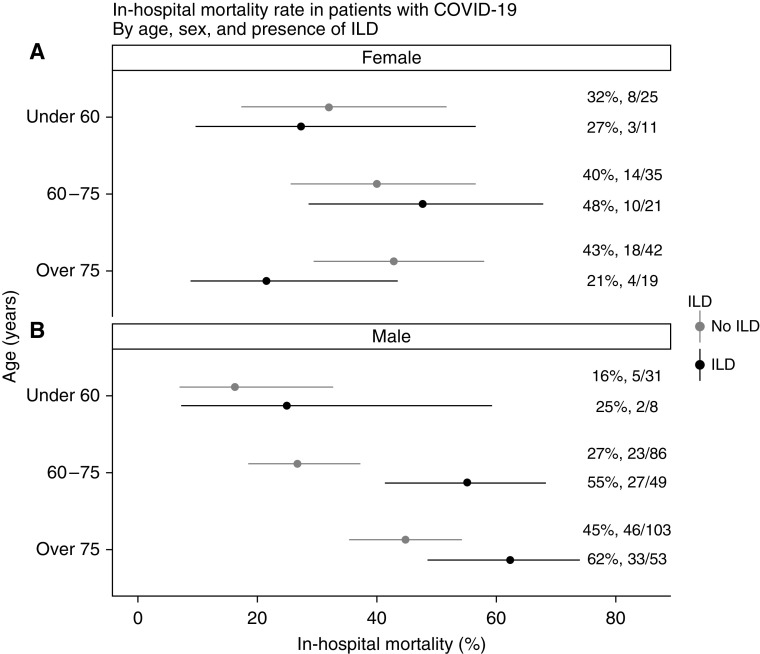
(*A*) In-hospital mortality with 95% confidence intervals for female patients with interstitial lung disease (ILD) hospitalized with coronavirus disease (COVID-19) stratified by age compared with those without ILD. (*B*) In-hospital mortality with 95% confidence intervals for male patients with ILD hospitalized with COVID-19 stratified by age compared with those without ILD.

**Figure 3.**
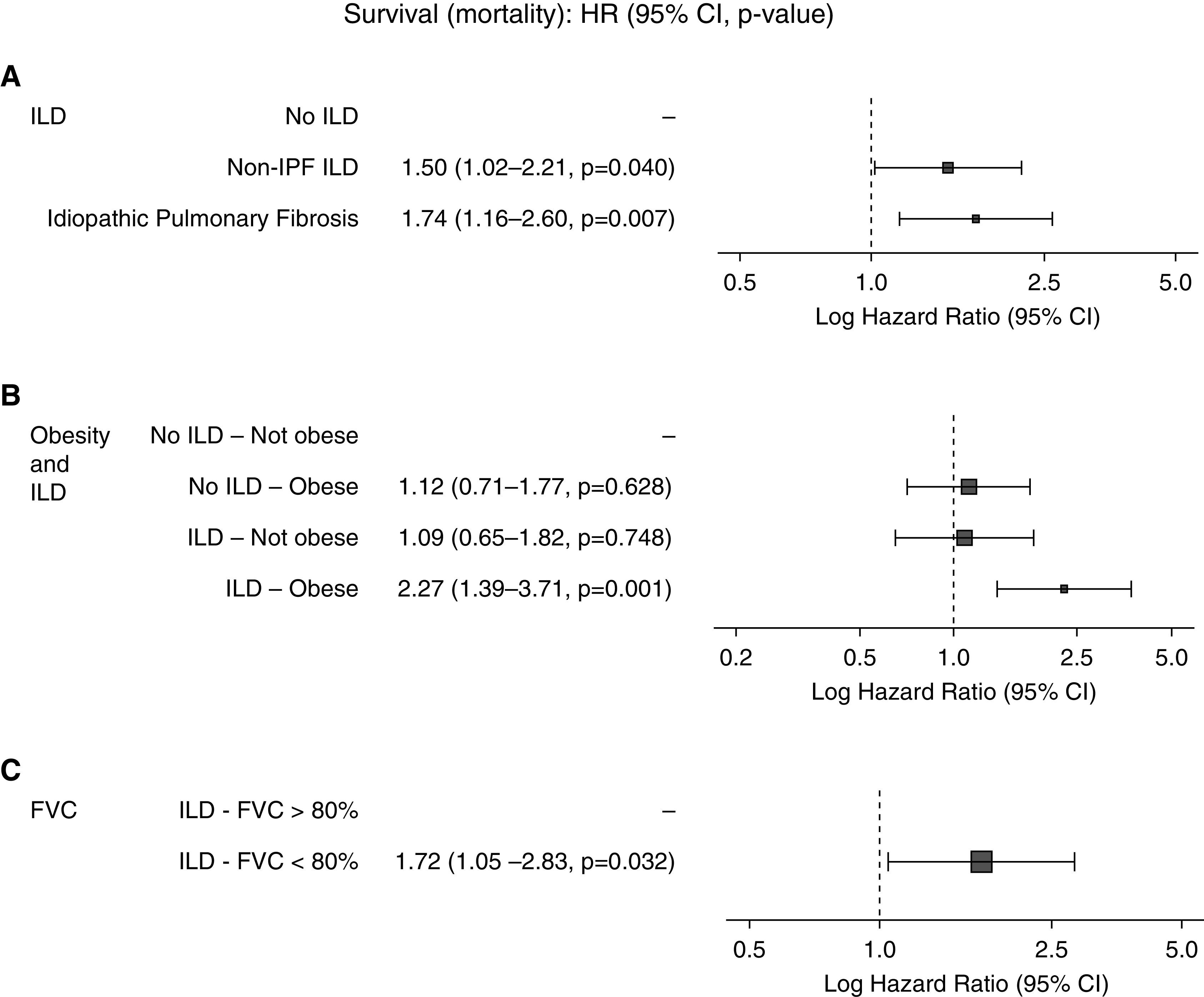
(*A*) Effect of interstitial lung disease (ILD) on mortality after coronavirus disease (COVID-19) (propensity matched), (*B*) effect of obesity and ILD (propensity matched), and (*C*) effect of FVC on outcome in patients with ILD only. CI = confidence interval; HR = hazard ratio; IPF = idiopathic pulmonary fibrosis.

After propensity score matching, 161 patients with ILD were compared with 322 patients admitted to the hospital between March 1 and May 1, 2020, but without ILD or other chronic lung disease (Table 1). There was significantly higher mortality in patients with ILD than in those without ILD (49% [79/161] vs. 35% [114/322]; *P* = 0.013), and ILD was associated with an increased risk of death in a matched adjusted analysis (HR, 1.60; 95% CI, 1.17–2.18; *P* = 0.003). There was significantly higher mortality in patients with ILD than in those without ILD (Figure 2), which was greatest in men and increased with age, which persisted for men after adjusting for age and comorbidity (adjusted HR, 1.98; 95% CI, 1.14–3.43; *P* = 0.015). Survival was significantly poorer in patients with IPF (HR, 1.74; 95% CI, 1.16–2.60; *P* = 0.007), and poorer survival was also seen in non-IPF ILD (HR, 1.50; 95% CI, 1.02–2.21; *P* = 0.040; Figure 3A) than in matched patients without ILD. Of patients with non-IPF ILD, those with chronic hypersensitivity pneumonitis and rheumatoid ILD had the highest mortality (50% [7/14] and 40% [4/10], respectively), whereas those with sarcoidosis and connective tissue disease (excluding rheumatoid)–related ILD had the lowest observed mortality (33% [3/9] and 23% [3/13], respectively). Overall, median length of stay in those who were still alive was not substantially different between patients without ILD (9 [IQR, 11] d) and those with non-IPF ILD (10 [IQR, 8] d) and IPF (10 [IQR, 8] d) (*P* = 0.725) (Table 2).

**Table 2.** Outcomes by ILD

	Total (*n*)	No ILD	ILD	*P* Value
Died	483	114 (35.4)	79 (49.1)	0.013
Respiratory support	483			
Unenhanced respiratory support*		254 (78.9)	136 (84.5)	0.288
Enhanced respiratory support		39 (12.1)	20 (12.4)	—
Ventilation		29 (9.0)	6 (3.7)	—
Length of stay for those alive, median (IQR)	290	9.0 (11.0)	10.0 (8.0)	0.725^†^

*Definition of abbreviations*: ILD = interstitial lung disease; IQR = interquartile range.

Data are shown as *n* (%) unless noted otherwise. All tests are chi-square except where noted.

* Standard-flow-rate oxygen supplementation not considered “enhanced” respiratory support; continuous positive airway pressure and high-flow O_2_ considered enhanced respiratory support.

^†^ Kruskal-Wallis used.

### Predictors of Outcome from COVID-19

Obesity has been previously described to increase the risk of death from COVID-19. After obesity was included in the propensity score matching (129/161 patients with ILD with data available, matched to 260 patients without ILD), being obese and having ILD was significantly associated with an increased hazard of death from COVID-19 (adjusted HR, 2.27; 95% CI, 1.39–3.71; *P* = 0.001; Figure 3B), which was greater than the effect of obesity in patients without ILD.

To determine whether patients with more severe ILD had a greater risk of mortality with COVID-19, the last available lung function results before hospital admission were analyzed. For all patients with ILD, the mean FVC in patients surviving COVID-19 was 82.2% predicted compared with 76.8% predicted in patients who died (*P* = 0.121). Similarly, the mean Dl_CO_ was 56.4% predicted in survivors compared with 49.6% in patients dying with COVID-19 (*P* = 0.072; Table 3). When the FVC was dichotomized using the 80% predicted threshold between mild and moderate ILD, in line with UK prescribing policy for antifibrotic medication, survival was significantly poorer in patients with moderate or severe ILD (HR, 1.72; 95% CI, 1.05–2.83) than in patients with mild disease (Figure 3C).

**Table 3.** Lung Function in ILD Group by Outcome

Label	Total (*n*)	Alive	Died	*P* Value
FVC, mean (SD)	147	2.7 (1.0)	2.6 (0.9)	0.372*
FVC, percentage of predicted, mean (SD)	151	82.2 (23.0)	76.8 (21.0)	0.135*
FVC, percentage of predicted	151			
<80%		35 (42.7)	42 (60.9)	0.039
>80%		47 (57.3)	27 (39.1)	—
FVC, percentage of predicted	151			
<50%		7 (6.9)	4 (4.8)	0.070
50–80%		28 (27.7)	38 (45.8)	—
80–100%		33 (32.7)	17 (20.5)	—
>100%		14 (13.9)	10 (12.0)	—
Dl_CO_, mean (SD)	81	5.2 (3.4)	4.2 (1.9)	0.093*
Dl_CO_, percentage of predicted, mean (SD)	122	56.4 (19.4)	49.6 (21.6)	0.072*

*Definition of abbreviation*: ILD = interstitial lung disease.

Data are shown as *n* (%) unless noted otherwise. All patients with ILD are shown, including those unmatched. All tests are Welch’s two-sample *t* test except where noted.

* Kruskal-Wallis used.

### Ventilatory Support for Patients with ILD with COVID-19

Most patients with ILD (84%; 135/161) did not receive enhanced respiratory support, which was similar to matched patients without ILD (79%; 254/322; Table 2). Significantly more patients who survived did not receive enhanced respiratory support (93%; 76/82) compared with those who died (75%; 59/79; *P* = 0.015). Of the 26 patients receiving enhanced respiratory support, 77% (20) died, including 83% (5/6) of matched patients with ILD receiving ventilation (Table 4).

**Table 4.** Use of Ventilation in Patients with ILD by Outcome

	Total (*n*)	Alive	Died	*P* Value
Respiratory support	161			
Unenhanced respiratory support*		76 (92.7)	59 (74.7)	0.015
Enhanced respiratory support		5 (6.0)	15 (23)	0.005
Ventilation		1 (1.2)	5 (3.8)	0.190

*Definition of abbreviation*: ILD = interstitial lung disease.

Data are shown as *n* (%). All tests are chi-square except where noted.

* Standard-flow-rate oxygen supplementation not considered “enhanced” respiratory support; continuous positive airway pressure and high-flow O_2_ considered enhanced respiratory support. Note that a further 10 patients with ILD were ventilated but not included in the matched cohort; of those, 6 survived.

### Effect of Antifibrotic and Immunosuppressive Therapy on Outcome from COVID-19

In patients with ILDs, 106 were taking no immunosuppressants or antifibrotics, of whom 50% died (53/106; Table 5). Almost one-third of patients with IPF (20/68) were receiving antifibrotic therapy at the time of admission. Seven patients were receiving pirfenidone and 13 were receiving nintedanib. Of those receiving antifibrotic therapy, 50% (10/20) died. Where immunosuppressants were reported, corticosteroids were most frequently prescribed (45/55) followed by mycophenolate (17/55); overall, 47% (26/55) of patients taking antifibrotics or immunosuppressants died. Significantly more patients with ILD received oral corticosteroids in the hospital than patients without ILD (27.8% vs. 9.9%; *P* < 0.001), but there was no difference in outcome between those receiving corticosteroids (48.9% mortality [22/55]) and those who did not (48.7% mortality [57/117]).

**Table 5.** Drugs Received by Patients with ILD in the Matched Cohort (*n* = 161)

	Alive	Died
Received immunosuppressants/antifibrotics, yes	29 (34.9)	26 (32.9)
Methotrexate/azathioprine, yes	3 (3.6)	2 (1.3)
Nintedanib, yes	9 (10.8)	4 (5.1)
Pirfenidone, yes*	1 (1.2)	6 (7.6)
Mycophenolate, yes	10 (12.0)	7 (8.9)
Corticosteroid, yes	23 (27.7)	22 (27.8)

*Definition of abbreviation*: ILD = interstitial lung disease.

Data are shown as *n* (%).

* Two further patients received pirfenidone and survived but were not included in the matched cohort.

## Discussion

Determining the risk of poor outcome in patients with preexisting conditions who acquire SARS-CoV-2 infection is crucial to determine what mitigation measures are required in the community. Our study shows that patients with existing interstitial lung disease were at very high risk of death when hospitalized with COVID-19, especially if they have reduced prior lung function or obesity. Most died without being offered mechanical ventilation; in those who were ventilated, mortality was very high.

Our observations were in keeping with other studies that have identified increasing age, comorbidities (diabetes mellitus, ischemic heart disease, and other non-ILD chronic pulmonary diseases), and male sex as predictors of poor outcome in COVID-19 (9, 12). Unfortunately, the risk for patients with ILD has not yet been quantified because patients with uncommon diseases often do not have their specific diagnostic information recorded in large observational studies or are often not accurately reported in administrative healthcare data sets. Despite the unknown risk of COVID-19 for patients with ILD, many patients chose to self-isolate, and therefore, there appeared to be apparent “protection” from COVID-19 observed to many ILD physicians around the world. To accurately understand the risk of COVID-19 and inform decision- and risk-matrices going forward, the European ILD community undertook an audit of hospitalized patients with ILD between the March 1 and May 1, 2020, to coincide with the first peak of COVID-19 within Europe. These data represent the largest assessment of the impact of SARS-CoV-2 infection on patients with ILD to date. These data demonstrate that patients with ILD are at increased risk of death after hospitalization for COVID-19, particularly elderly males with poor lung function and obesity. It is notable that the risk of in-hospital mortality from COVID-19 for patients with ILD is particularly associated with male sex and obesity. These risk factors are associated both with development of IPF and with mortality from COVID-19 in the general population, which may suggest synergy between shared mechanisms of pathogenesis (13).

The role of viral infection promoting acute exacerbations of ILD has been investigated for a number of years without a definitive answer, which probably reflects the range of viruses studied, the number of patients needed to explore such a hypothesis, and more broadly the challenges studying acute exacerbations of ILD (14–17). These data confirm that patients with ILD are at increased risk of mortality from COVID-19 compared with a matched population without ILD, and the risk is greatest for IPF consistent with a respiratory virus inducing acute exacerbations of ILD.

Factors associated with poor prognosis in acute exacerbations of ILD include pulmonary fibrosis and poor lung function before admission and fibrotic ILDs (18–21). The data presented here support these prior studies demonstrating that patients with a recorded FVC <80% predicted before admission had an increased mortality compared with those with an FVC >80%. Similarly, patients with fibrotic ILDs such as chronic hypersensitivity pneumonitis had mortality rates comparable with IPF and higher than those with sarcoid or connective tissue disease–associated ILD. Interestingly, obese patients with ILD had a substantially increased risk of mortality. This may reflect use of steroids in severe disease, although we do not favor this hypothesis as there was no apparent increased risk of death associated with corticosteroid use. Obesity may be due to limited activity in patients with ILD, a condition that leads to progressive reduction in exercise tolerance as it becomes more severe (22). Alternatively, and perhaps most intriguingly, it may lend support to the hypothesis that severe ILD is part of the “metabolic syndrome” (23).

This analysis also assessed respiratory support offered to patients hospitalized with ILD. In line with current practice and guidelines, most patients did not receive any ventilatory support (7, 24). Most patients who survived did not require enhanced respiratory support such as high-flow oxygen, continuous positive airway pressure, or ventilation, and of those receiving enhanced respiratory support, the majority died. These data continue to support the use of supportive care in preference to either noninvasive or invasive mechanical ventilation except in clearly defined cases such as predominantly inflammatory ILD or bridging to transplantation.

This analysis has a number of strengths. It is the largest international, multicenter study to assess the outcome of patients with interstitial lung disease hospitalized with the respiratory infection SARS-CoV-2. Merging of the ILD Audit data set with the ISARIC4C CCP-UK data set enabled accurate, contemporary propensity score matching for a number of potential confounding factors to assess the risk from COVID-19 disease. This enabled an assessment of the risk to patients with ILD infected with SARS-CoV-2, facilitating evidence-based mitigation strategies, such as self-isolation, for this vulnerable group of patients.

This study also has some potential weaknesses. Because of the retrospective nature of the data collection, recall bias might have led to overselection of severe cases of COVID-19 in patients with ILD; however, this is mitigated by the large number of centers participating in the audit. Similarly, because only hospitalized patients could be included, it is possible that a large number of patients with ILD and COVID-19 were omitted, and therefore, the risk of COVID-19 could be overstated. However, given the demographic associated with ILD, we think this is unlikely. Also, the comparator group only included patients from the UK and some younger patients, which could have led to residual confounding by geographical determinants of disease severity and treatment; however, because 86% of the patients with ILD were recruited from the UK, we think this is unlikely to be a major source of confounding. Although patients with ILD were less likely to receive invasive mechanical ventilation, this is more likely due to their severe respiratory disease; however, there was insufficient detail in the audit data collection to match for COVID-19 severity based on admission severity scores or provide more granularity relating to the nature of respiratory support received. However, we did not observe any patterns across the groups that were likely to have an effect. Similarly, the propensity score matching will have helped to address any imbalances that are observed within the matching variables.

The effect of obesity was assessed in the matched population, suggesting an increased risk in patients without ILD; however, in contrast with prior reports (9, 25), no effect of obesity was observed in the control population. This reflects the limitations associated with recording obesity using inpatient data where obesity is not objectively measured as well as the relatively small numbers of patients used in the matching and the large amount of missing data relating to weight. Therefore, the data relating to obesity must be interpreted accordingly, but we believe as a modifiable risk factor it is important to highlight the risk of obesity in patients with ILD who might develop COVID-19. Finally, it was not possible to evaluate specific treatment effects, such as the use of antifibrotics, immunosuppressants, or antiviral therapies. The audit was undertaken in the early part of the pandemic when hydroxychloroquine and remdesivir were only available in the context of clinical trials. The UK Medicine Health Regulatory Agency did not approve remdesivir for use in the UK until May 26, 2020, and hydroxychloroquine has been shown to have no effect on outcome in any circumstance of COVID-19 infection (26, 27); therefore, it is highly unlikely that the results are confounded by antiviral therapy. Only a proportion of patients received antifibrotics during their admission. Furthermore, owing to the unknown effect of antifibrotics and concerns regarding immunosuppression on the clinical course of COVID-19, there is likely to be considerable confounding owing to indication. There are both potential benefits (28, 29) and harms associated with background therapy for ILD (30). With specific reference to corticosteroids, our analysis did not show any effect on mortality in patients with ILD. Although the RECOVERY (Randomised Evaluation of COVID-19 Therapy) study demonstrated an overall beneficial effect of dexamethasone in patients with COVID-19, there was considerable heterogeneity in the response (27), and it is not clear to what extent steroid choice, steroid dosing, steroid duration, or pre–COVID-19 steroid therapy impact on outcome. However, it is reassuring from a safety perspective that there was no obvious signal suggesting harms associated with background therapy for ILD in patients with COVID-19, although further study to evaluate the consequence of antifibrotic and immunomodulatory therapy in ILD are needed.

### Conclusions

These data demonstrate that patients with ILD, particularly those with fibrotic ILD, are at higher risk of mortality from COVID-19 than patients without ILD. Furthermore, the risk is heightened in elderly males and those with obesity or poor lung function, and we would recommend dietary advice in overweight patients. We also propose that patients with ILD, particularly severe fibrotic ILD, continue to be regarded as high risk of mortality from COVID-19 and follow national self-isolation guidelines for vulnerable individuals and be prioritized for SARS-CoV-2 vaccination at such time as it becomes available. Finally, we believe these data demonstrate the importance of international collaboration to collect data to understand the consequences of emerging threats to patients with ILD.

## Supplementary Material

Supplements

Author disclosures
