# Supplementary material for: Outcome of Hospitalization for COVID-19 in Patients with Interstitial Lung Disease. An International Multicenter Study
Source: Am J Respir Crit Care Med. 2020 Dec 15;202(12):1656–65. doi: 10.1164/rccm.202007-2794OC (PMC7737581; doi:10.1164/rccm.202007-2794OC)
Supplement: Supplements [file rccm.202007-2794OC.html]

Outcome of Hospitalization for COVID-19 in Patients with Interstitial Lung Disease. An International Multicenter Study | American Journal of Respiratory and Critical Care Medicine

- disclosures.pdf (5 MB)
- drake\_data\_supplement.pdf (868 KB)
