# Supplementary material for: Outcome of Hospitalization for COVID-19 in Patients with Interstitial Lung Disease. An International Multicenter Study
Source: Am J Respir Crit Care Med. 2020 Dec 15;202(12):1656–65. doi: 10.1164/rccm.202007-2794OC (PMC7737581; doi:10.1164/rccm.202007-2794OC)

Outcome of hospitalization for COVID-19 in patients with Interstitial Lung Disease: An international multicenter study.

Thomas M Drake, Annemarie B Docherty, Ewen M Harrison, Jennifer K Quint, Huzaifa Adamali, Sarah Agnew, Suresh Babu, Christopher M Barber, Shaney Barratt, Elisabeth Bendstrup, Stephen Bianchi, Diego Castillo Villegas, Nazia Chaudhuri, Felix Chua, Robina Coker, William Chang, Anjali Crawshaw, Louise E. Crowley, Davinder Dosanjh, Christine A Fiddler, Ian A. Forrest, Peter George, Michael A Gibbons, Katherine Groom, Sarah Haney, Simon P Hart, Emily Heiden, Michael Henry, Ling-Pei Ho, Rachel K Hoyles, John Hutchinson, Killian Hurley, Mark Jones, Steve Jones, Maria Kokosi, Michael Kreuter, Laura MacKay, Siva Mahendran, George Margaritopoulos, Maria Molina-Molina, Philip L Molyneaux, Aiden O'Brien, Katherine O'Reilly, Alice Packham, Helen Parfrey, Venerino Poletti, Joanna C. Porter, Elisabetta Renzoni, Pilar Rivera-Ortega, Anne-Marie Russell, Gauri Saini, Lisa G Spencer, Giulia M. Stella, Helen Stone, Sharon Sturney, David Thickett, Muhunthan Thillai, Tim Wallis, Katie Ward, Athol U Wells, Alex West, Melissa Wickremasinghe, Felix Woodhead, Glenn Hearson, Lucy Howard, J Kenneth Baillie, Peter J.M. Openshaw, Malcolm G Semple, Iain Stewart, ISARIC4C Investigators, R Gisli Jenkins

ONLINE DATA SUPPLEMENT

Outcome of COVID-19 in patients hospitalized with Interstitial Lung Disease: An international multicenter study. Supplementary Material.

Supplementary Table E1

COUNTRY	NUMBER	PERCENTAGE
**UK**	299	86
**REPUBLIC OF IRELAND**	11	3
**SPAIN**	27	7.5
**GERMAN**	5	1.5
**ITALY**	5	1.5
**DENMARK**	<3	<1

Supplementary Table E2

label	'Other' ILD diagnoses	n
Other ILD Details	Age-related ILD/presbyotic lung	<3
	ANCA vasculitis	<3
	asbestosis	5
	Autoimmune Pneumonitis	5
	Chronic eosinophilic pneumonia	<3
	Combined Emphysema and Pulmonary Fibrosis	7
	Desquamative Intestitial Pneumonia	<3
	Non-Specific Interstitial Pneumonia	10
	Organising Pneumonia	<3
	PPFE	<3
	smoking related ILD	<3
	Unclassifiable ILD	11

Supplemental Figure

Figure E1 Balance Plots

Distribution of differences across ILD groups by matching variable. Propensity score matching normalised standardized mean differences across the treatment groups, with observed differences before matching not being present after.

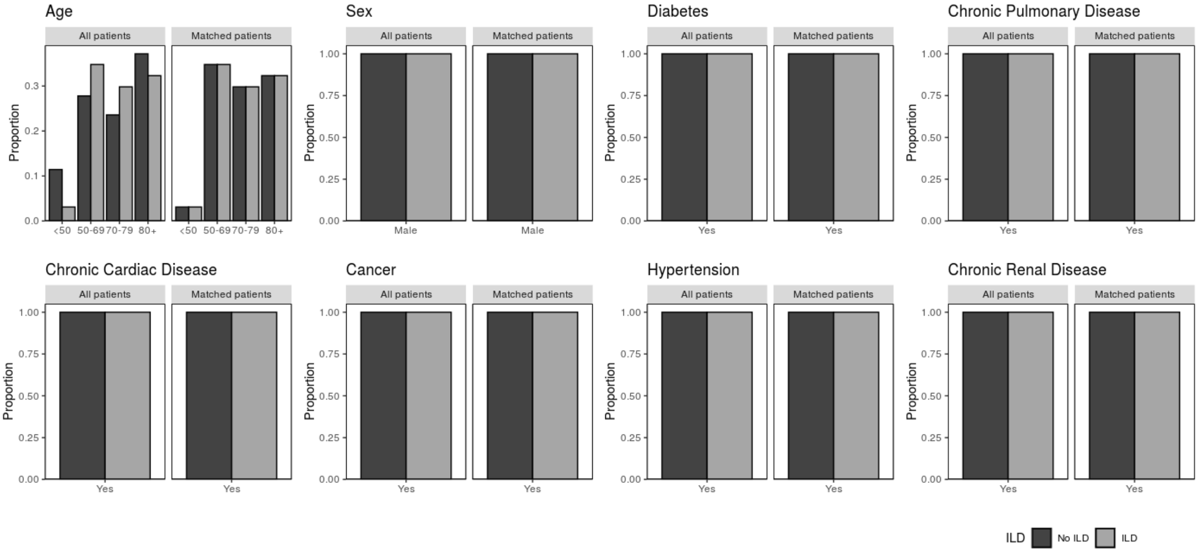

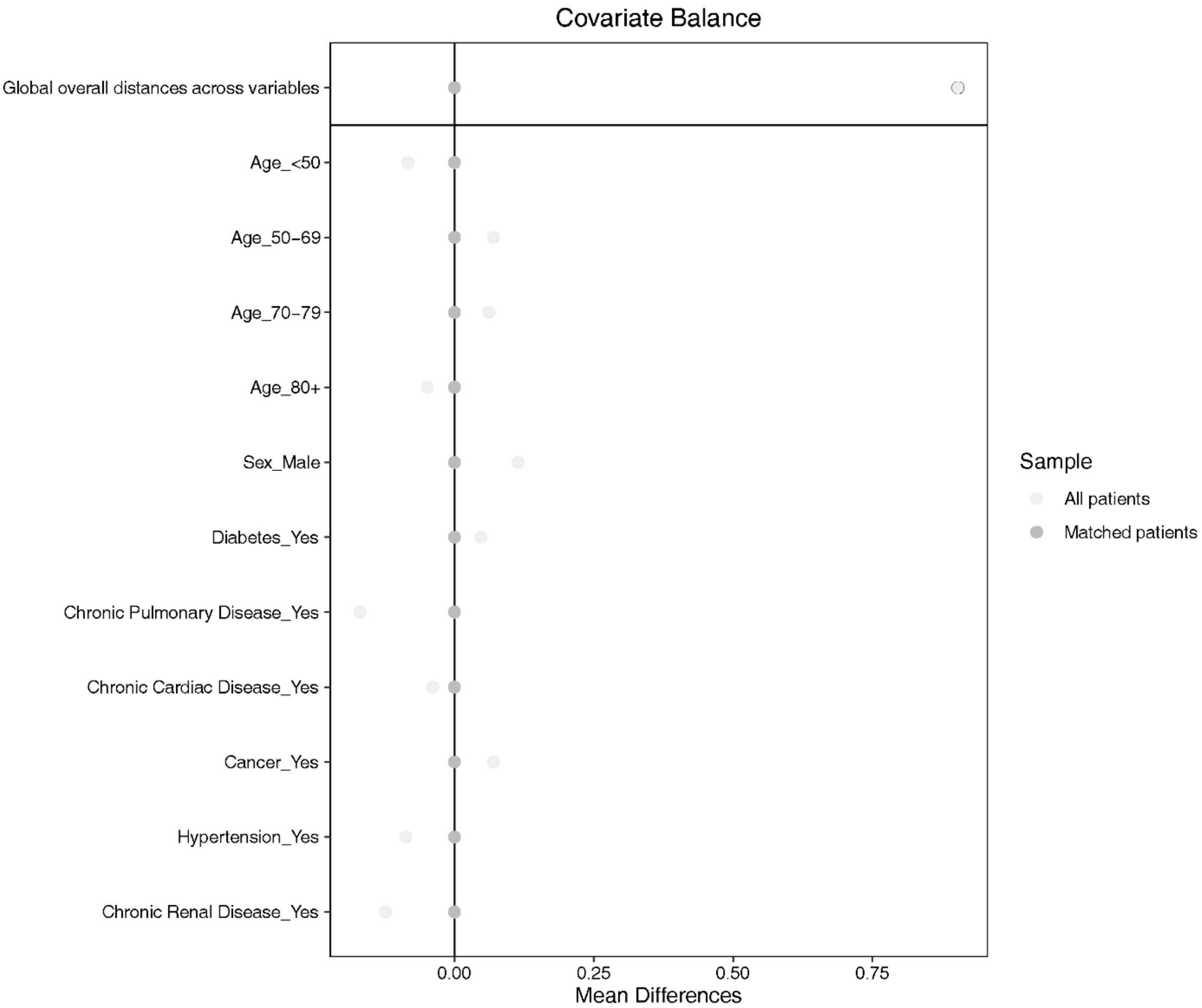

Figure E2 Impact of sex, and co-morbidity on outcomes of COVID-19 in ILD]

Survival (mortality): HR (95% CI, p−value)

Age (per year increase)	–	1.01 (0.99−1.02, p=0.565)	
Sex at Birth	Female	–	
	Male	1.98 (1.14−3.43, p=0.015)	
Chronic Cardiac Disease	No	–	
	Yes	0.98 (0.60−1.60, p=0.942)	
Diabetes	No	–	
	Yes	1.24 (0.77−2.00, p=0.377)	
Hypertension	No	–	
	Yes	0.95 (0.60−1.50, p=0.817)	

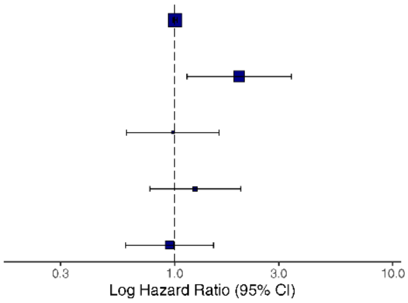

Supplement: Supplements [file rccm.202007-2794OC_drake_data_supplement.pdf]
